# Pulmonary rehabilitation improves sleep efficiency measured by actigraphy in poorly sleeping COPD patients

**DOI:** 10.1038/s41598-023-38546-3

**Published:** 2023-07-13

**Authors:** Maria Gabrovska, Audrey Herpeux, Anne-Violette Bruyneel, Marie Bruyneel

**Affiliations:** 1 Department of Pulmonary Medicine, CHU Saint-Pierre, Rue Haute, 322, 1000 Brussels, Belgium; 2 Université Libre de Bruxelles (ULB), Brussels, Belgium; 3 Geneva School of Health Sciences, HES-SO School of University of Applied Sciences and Arts Western Switzerland, Geneva, Switzerland

**Keywords:** Outcomes research, Respiratory tract diseases, Diseases

## Abstract

Chronic insomnia is reported by up to 50% of chronic obstructive pulmonary disease (COPD) patients. This may be attributable to several factors including nocturnal dyspnea, reduced physical activity, and less time outside. Pulmonary rehabilitation (PR) is recommended in COPD to improve both physical and psychological conditioning. The aim of this study was to assess the effect of PR on sleep efficiency (SE, measured by actigraphy) in COPD patients. COPD eligible for PR were prospectively included. Baseline and post PR (30 sessions) assessments included incremental and maximal exercise testing, 6-min walking distance test (6MWT), actigraphy, and questionnaires [Pittsburgh Sleep Quality Index (PSQI), Hospital Anxiety Depression scale, St George Respiratory, and modified Medical Research Council dyspnea scale]. Sixty-one patients were included, and 31 patients completed the study protocol (68% of males, age 63 ± 9 y, FEV1 44.2 ± 12.3%). After PR, SE remained unchanged, p = 0.07, as well as PSQI score (p = 0.22), despite improvements in exercise capacity (incremental exercise test, 6MWT) and dyspnea. However, SE improved significantly in the poor sleeper subgroup (SE < 85%, n = 24, p = 0.02), whereas the PSQI remained unchanged. The present study shows, in COPD patients included in a PR program, that improvement in exercise capacity was disappointingly not associated with a better SE assessed by actigraphy. Subjective sleep quality was also unchanged at the end of PR program. However, SE improved significantly in the poor sleeper subgroup (SE < 85%). Further studies are required to better characterize the origin of sleep disturbances in COPD and the potential benefit of some (non-)pharmacologic interventions.

## Introduction

Chronic obstructive pulmonary disease (COPD) patients often complain of chronic insomnia, with up to 50% of patients reporting that they have difficulty falling asleep, staying asleep, or they have unrefreshing sleep^1,2^.This prevalence is double that of the general population and may be attributable to several factors, including nocturnal dyspnea, reduced physical activity and time spent outside, reduced exposure to bright light, and expansion of sleep opportunity as a means of coping with disease-related fatigue^3^. Nocturnal dyspnea and COPD-related medication can further deteriorate sleep. Depression and anxiety are also contributors to poor sleep and these are very prevalent in COPD, with rates of 25% and 40%, respectively^4^.

Recently, a relationship between low activity levels and sleep disturbance in COPD has been demonstrated using actigraphic measurements^5^. It is not currently known whether poor sleep induces low activity levels or the opposite in COPD. However, it has been shown in insomniacs that increasing physical activity (PA) improves sleep quality^6^.

Pulmonary rehabilitation (PR) is a recommended comprehensive intervention in COPD that is based on a thorough patient assessment followed by patient-tailored therapies that include exercise training, education, and behavioral changes designed to improve both physical and psychological condition and to promote long-term adherence to health-enhancing behaviors^7^. PR has been shown to decrease anxiety and depression, improve quality of life, and reduce healthcare costs and hospitalizations^8,9^. In addition, PR has recently been shown to be associated with improvements in both subjective sleep quality and daytime sleepiness in a small series of COPD patients^10^.

The primary aim of this study was to assess the effect of PR on sleep efficiency (SE) in COPD patients. Secondary aims included the effect of PR on subjective sleep quality, quality of life, anxiety and depression, and PA.

## Methods

### Patients

Adult patients with moderate-to-severe COPD who were eligible for PR were prospectively included. Indication for PR was established according to ERS/ATS guidelines^7^ and to national social security rules. Exclusion criteria were as follows: inability to perform PR (neurologic/orthopedic disability), inability to perform measurements for cognitive or language concerns, known or suspected obstructive sleep apnea syndrome, duration of PR (30 sessions) exceeding 5 months.

The study protocol was approved by the Saint-Pierre University Hospital ethics committee (B076201734220). All procedures performed in studies involving human participants were in accordance with the ethical standards of the institutional and/or national research committee and with the 1964 Helsinki declaration and its later amendments or comparable ethical standards. All included patients provided written informed consent to participate in the study.

### Study design

This was a prospective observational study. Sleep and exercise were assessed at baseline and at the end of the PR program.

### Baseline measurements

#### Exercise capacity

Incremental and maximal exercise testing was conducted on an electronically-braked cycle ergometer, to a symptom-limited endpoint, with a Jaeger ergospirometry system OxyconPro (CareFusion), and LabManager V5-32.0 software, according to American guidelines^11^.

The 6-min walk test (6MWT) with continuous SpO2 monitoring and heart frequency was also performed^12^.

#### Actigraphy

Objective activity and energy expenditure were evaluated using the Bodymedia SenseWear Pro Armband® (SWA) activity monitor^13^. Built-in sensors collected data for accelerometer (3-axis), heat flux, skin temperature, and galvanic skin response in 1-min epochs. The activity monitor was attached to an adjustable Velcro armband worn on the non-dominant upper arm for 7 days. Subjects were instructed to wear it around-the-clock with the exception of activities (ie, showering) that could get the activity monitor wet. Data analyses were performed on 5 days (one weekend day and 4 week days). The manually selected days correspond to days with data obtained from midnight to midnight and where the wearing compliance was maximal. We have chosen to analyze several week days and one weekend day in order to reflect not only the usual sleep patterns of the patients, as these can differ from week days to weekend days^14^ but also to achieve reliability in the physical activity estimate^15^. The analysis of the selected days was made automatically by SWA algorithm. The activity level was expressed as the mean daily activity count for steps walked knowing that a middle age free-living adult walks about 8500 steps/day^16^ and COPD patients about 3500 steps/day^17^. Activity was also assessed through measurement of total estimated energy expenditure and mean metabolic equivalent of tasks (METs). Sedentary activity is considered for < 3 METs, moderate for 3–5.9 METs and vigorous for ≥ 6 METs. Actigraphy was also used to record total sleep time (TST), time in bed (TIB) and SE since it can reliably measure these parameters^18^. Indeed, actigraphy has been shown to be accurate to estimate sleep^19^. SE < 85% is considered to be a sign of insomnia^20^.

#### Questionnaires

Sleep quality and quantity was assessed subjectively by using the Pittsburgh Sleep Quality Index questionnaire (PSQI)^21^. The PSQI includes seven components of subjective sleep: sleep quality, sleep latency, sleep duration, sleep efficiency, sleep disturbance, the use of sleep medications, and daytime dysfunction. The overall score ranges from 0 to 21, with higher scores indicating poor quality sleep and scores less than 5 considered to be high-quality sleep. The Hospital Anxiety Depression scale (HADS) was used to identify depression and anxiety among patients in nonpsychiatric care settings^22^. This questionnaire includes an anxiety subscale (HADS-A) and a depression subscale (HADS-D) with 14 mixed items. Each item is rated on a four-point scale (0–3), yielding maximum scores of 21 for anxiety and depression. Scores of more than 11 on either subscale indicate significant psychological comorbidity, a score of 8–10 is borderline, and a score of 7 or below is considered to be normal.

Quality of life was assessed through the St George respiratory questionnaire (SGRQ)^23^. This questionnaire includes two parts, Symptoms and Activity / Impacts. Scores range from 0 to 100, with higher scores indicating more limitations. The minimal clinically important difference is a decrease of 4 units^24^. Breathlessness was scored using the modified Medical Research Council (mMRC) dyspnea scale. A decrease of one point is considered to be clinically significant^25^.

#### Pulmonary rehabilitation program

The outpatient PR program included 30 group sessions, organized three times per week. This pluridisciplinary program follows the recommendations of ATS/ERS and includes interventions from a pulmonologist, physiotherapists, and an occupational therapist, and advice from a dietician and a tobacco specialist-psychologist^7^.

Physical training is adapted to the patient's own capacities and evolves according to the patient's symptoms and improvement. It consists of endurance training and muscular reinforcement training. At each session, all patients completed 30 min of interval-training cycling using a 2–4 min interval, at low load for 4 min, slightly below the ventilatory threshold previously measured during ergospirometry, then at high load for 2 min, set at around 85% of the maximum load achieved during the same maximal test. In addition, most patients used the treadmill in continuous mode for 10 min, adjusting the slope and speed of the treadmill to obtain a walking intensity greater than the patient's usual walking rhythm. Some patients who were sufficiently comfortable on the treadmill used the 2–2 min training interval, varying the speed. The steps were also designed to create the equivalent of 60 steps. Each session included a muscular reinforcement training targeting the biceps and deltoids of the upper limbs, the pectoral and dorsal muscles, and the quadriceps of the lower limbs, using dumbbells and weight machines, by performing 3 sets of 10 repetitions for each muscle group worked. Alongside this physical training, patients benefit from therapeutic education sessions and psychological support. At the end of PR, the baseline measurements were repeated.

### Definition of sleep quality

Sleep quality refers to a combination of subjective parameters of initiation and maintenance of sleep, assessed by questionnaires^26^. Objective sleep quality is based on several parameters such as sleep latency, wake after sleep onset, TST and SE, measured by polysomnography (PSG)^27^ or surrogate, e.g. actigraphy^28^. Subjective sleep quality is closely related to SE measure by PSG, but not to sleep stages^29^. On the basis of these data, we focused on this parameter to approach the objective measurement of sleep quality.

### Statistical analysis

Analyses compared the results for each variable between baseline measurement and post-rehabilitation measurement. Descriptive statistics, comprised of mean and standard deviation (SD), were used to define each data acquisition time point (baseline and post-rehabilitation). The Shapiro–Wilk test was applied in order to confirm the data distribution and to determine the correct statistical tests. Paired t-tests were performed to examine pre- and post-rehabilitation differences. A p value of < 0.05 was considered to indicate a statistically significant difference between the time points. All statistical analyses were performed using SPSS© (IBM analytics, V.25, 2017).

### Ethics approval and informed consent

The study protocol was approved by the Saint-Pierre University Hospital ethics committee (B076201734220). All procedures performed in studies involving human participants were in accordance with the ethical standards of the institutional and/or national research committee and with the 1964 Helsinki declaration and its later amendments or comparable ethical standards. All included patients provided written informed consent to participate in the study.

## Results

Between November 2017 and December 2021, 74 patients were screened and 61 successfully included. 31 patients completed the study protocol. Drop-out were related mainly to COPD exacerbations, other acute diseases, death, poor compliance to PR program and PR program lasting more than 5 months.

**Figure 1:**
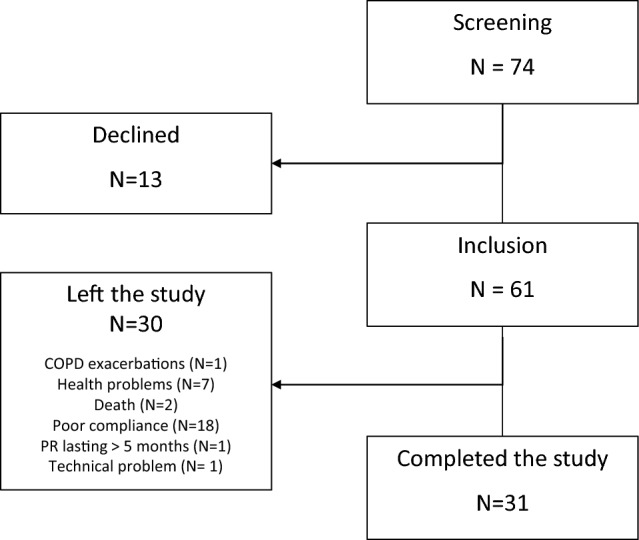
Flow chart of the study. COPD: chronic obstructive pulmonary disease, PR: pulmonary rehabilitation.

Flow chart is shown on Fig. 1.

Patient’s characteristics and demographics are reported in Table 1.

**Table 1:** patient’s characteristics and demographics.

	Mean ± SD
Age (years)	63 ± 9
Sex	
M (%)	68
F (%)	32
BMI (kg/M^2^)	24 ± 5
Spirometry	
FEV1/FVC (%)	45.9 ± 11.9
FEV1(% pred.)	44.2 ± 12.3
FVC (% pred.)	75.9 ± 16.7
TLC (% pred.)	113.9 ± 19.3
RV/TLC (%)	57 ± 7.4
DLCO (% pred)	50.7 ± 15.3
Smoking history (%)	
Former smokers	74
Current smokers	26
Pack-year	60 ± 25
Comorbidities (%)	
Cardiovascular diseases	48.4
Anxiety/depression	41.9
Metabolic disorders	35.5
Osteoporosis	12.9
Medication (%)	
LAMA	3.2
LABA	3.2
LABA-LAMA	64.5
LABA-ICS	3.2
LABA-LAMA-ICS	25.8
Hypnotics	6.4
Anxiolytics	6.4

BMI: body mass index, LABA: long acting beta agonist, LAMA: long acting muscarinic antagonists, ICS: inhaled corticosteroids, DLCO: diffusing capacity of the lungs for carbon monoxide, FEV1: forced expiratory volume in one second, SD: standard deviation, % pred.: percent of predicted value. Cardiovascular diseases.

Include stroke, hypertension, cardiomyopathy, arrhythmia. Metabolic disorders include dyslipidemia and diabetes mellitus. Hypnotics refers to benzodiazepines and Z-drugs.

All the patients completed the PR (mean: 30.3 ± 2.7 sessions). At the end of PR, mean exercise capacity increased on incremental exercise test, 6MWT and number of steps walked/day were significantly improved. (Table 2).

**Table 2:** Patient’s activity and sleep assessment.

Variable	Baseline	Post-PR	p
Incremental cardiopulmonary exercise test			
Work load peak (W)	71.1 ± 22.0	80.1 ± 24.7	< 0.01
Workload AT (W)	38.6 ± 14.4	48.6 ± 15.3	< 0.01
Workload AT/workload peak (%)	54.6 ± 13.7	61.5 ± 10.7	< 0.01
VO2 peak (l/min)	1.0 ± 0.3	1.1 ± 0.3	< 0.01
VO2 AT(l/min)	0.7 ± 0.2	0.8 ± 0.2	< 0.01
VO2 AT/VO2 peak (%)	70.3 ± 12.0	73.6 ± 11.7	0.15
6MWT			
Distance (m)	459.9 ± 85.2	480.5 ± 82.6	< 0.01
Actigraphy			
TST (min)	411.3 ± 112.2	423.1 ± 103.9	0.42
SE (%)	78.6 ± 8.1	81.5 ± 6.5	0.07
MET	1.36 ± 0.28	1.40 ± 0.21	0.36
Number of steps walked/day	3832.2 ± 2552.8	4796.5 ± 2840.3	0.03
Sedentary physical activity (0–2.9 METs) (min)	1296.4 ± 148.2	1292.0 ± 95.2	0.80
Moderate physical activity (3–5.9 METs) (min)	97.3 ± 81.7	110.1 ± 65.1	0.37
Vigourous physical activity (6-9METs) (min)	1.9 ± 4.2	2.9 ± 4.6	0.08
Questionnaires			
SGRQ symptoms	33.8 ± 17.8	30.0 ± 20.0	0.20
SGRQ activity	62.8 ± 17.6	57.6 ± 21.0	0.02
SGRQ impact	26.7 ± 18.3	25.8 ± 19.1	0.67
SGRQ global score	38.9 ± 15.5	36.2 ± 17.6	0.10
HADS-A	6.5 ± 2.9	6.8 ± 3.3	0.61
HADS-D	4.5 ± 3.6	4.6 ± 3.9	0.90
PSQI (total score)	7.4 ± 4.5	6.6 ± 4.3	0.22
Subjective quality of sleep	6.9 ± 4.3	6.5 ± 4.7	0.60
Sleep latency	1.0 ± 0.8	1.1 ± 1.0	0.41
Sleep duration	1.5 ± 1.3	1.2 ± 1.2	0.38
Habitual sleep efficiency	1.1 ± 1.2	1.0 ± 1.0	0.62
Sleep alterations	0.9 ± 1.2	1.0 ± 1.2	0.57
Use of sleep medications	1.3 ± 0.5	1.1 ± 0.6	0.09
Daytime sleep dysfunction	0.6 ± 1.1	0.7 ± 1.2	0.37
mMRC	0.7 ± 0.8	0.4 ± 0.8	0.06

AT : anaerobic threshold, VO2 : oxygen uptake, TST : total sleep time, SE : sleep efficiency, 6MWT : 6-min walk test, MET : metabolic equivalent of task, PR: pulmonary rehabilitation, SGRQ: St George respiratory questionnaire, HADS: hospital anxiety and depression scale, PSQI: Pittsburgh Sleep Quality Index questionnaire, mMRC: modified medical research council.

Prevalence of poor sleep quality based on actigraphy measured SE < 85% was 77% (24/31) and based on PSQI ≥ 5 was 61% (19/31).

On actigraphy, no significant increase in SE was observed. Subjective sleep quality, based on PSQI score (total or for the separate components), remained also unchanged (Table 2).

**Figure 2:**
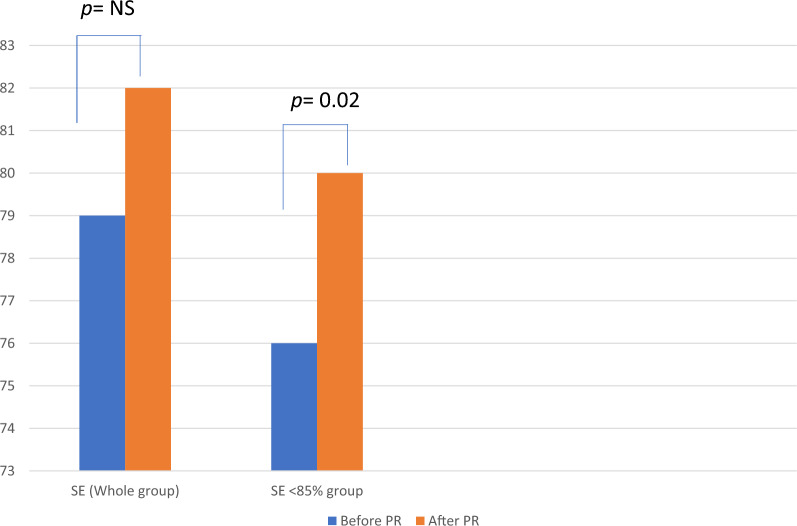
Changes in sleep efficiency after pulmonary rehabilitation, for the whole group (n = 31) and for the group with baseline reduced sleep efficiency (n = 24). SE: sleep efficiency, PR: pulmonary rehabilitation, NS: statistically non significant difference, *p:* p value.

However, in the 24 patients with a reduced SE at baseline, SE increased significantly after PR completion, from 76.00 ± 6.86 to 80.42 ± 6.21, p = 0.02 (Fig. 2), despite no significant change in PSQI total score (p = 0.44).

In the 19 patients showing high PSQI score baseline, we did not report any significant change in SE or PSQI after PR. When analyzing separate components, a significant improvement was observed for “Daytime sleep dysfunction”, p = 0.01, but not for the others.

Regarding anxiety and depression (HADS-A and HADS-D), no change was observed after PR.

Quality of life measured by SGRQ showed an improvement in item “activity”. Questionnaires results are summarized in Table 2.

## Discussion

The present study demonstrates that participation in a pulmonary rehabilitation (PR) program did not result in improved subjective and objective sleep efficiency (SE) among a group of moderate-to-severe COPD patients. This finding contradicts previous studies that showed improvements in subjective SE as measured by the Pittsburgh Sleep Quality index (PSQI) after completing PR^10,30,31^.

However, a significant increase in objective SE, measured through actigraphy, was observed in a subgroup of COPD patients with poor sleep at baseline (SE < 85%) in this study. In these patients, subjective sleep quality remained unchanged, contrarily to previous reports, showing PSQI improvements after PR completion.

Indeed, subjective sleep improvements have been described in chronic cardiopulmonary disorders following specific interventions. For example, Rouleau et al. described sleep symptoms in 80 patients with cardiovascular disease following a cardiac rehabilitation program. At baseline, insomnia symptoms, assessed by the Insomnia symptom severity scale, were present in 40% of patients. This proportion was significantly reduced after 12 weeks of cardiac rehabilitation^32^. In COPD, subjective sleep quality, assessed by PSQI, improved in 3 out of 4 studies after PR^10,30,31,33^.

But regarding objectively measured sleep quality, measured by actigraphy, no improvement was observed in a group of 48 COPD after PR^28^. A recent retrospective study in a larger sample of COPD did also not show any improvement in actigraphic sleep parameters^34^. However, in that study, significant subjective improvement was observed on PSQI score in poor sleepers (baseline PSQI ≥ 5). In contrast to this study, we only observed an improvement in objective, not subjective, sleep quality in the poor sleeper subgroup. This may be linked to several factors. Patients were slightly older, and a higher proportion of patients complained of poor sleep on the PSQI (85% vs. 61% in the present study). This observation can be related to the physiological decrease of SE and total sleep time with aging^35^. Moreover, PR program was shorter, 8 weeks. Indeed, longer PR programs are thought to produce greater gains^36^. In addition, the authors did not analyze patients with poor sleep on the basis of actigraphic parameters. Nor was this the case in the study by Cox et al.^28^.

Other factors than limited exercise capacity and poor PA are certainly contributing to poor sleep in COPD. Indeed, in our series, 42% suffered from anxiety and/or depression and 26% were still active smokers. Anxiety, depression, COPD-related medication, cough, smoking status and age have been identified as factors negatively affecting sleep^37^. These factors could explain the absence of improvement in PSQI scores despite an objective effect of PR on SE in poor sleepers.

In the present study, no improvement of anxiety and depression occurred, contrarily to previous reports^9^, but the scores HADS scores were normal at baseline.

We also confirmed the good results of patients in terms of exercise tolerance for the patients able to adhere and participate to the whole program. As presented in the flow chart, this beneficial comprehensive individualized program is often abandoned by the patients. Large drop-out rates (49% in the present study) have been previously observed in other series, with 29–60% of patients who do not complete the PR program^38–40^. Factors such as current smoking, poor shuttle walking distance and hospitalizations have been incriminated. Additionally, 2 of our patients died during the PR, emphasizing the frailty of PR population.

### Limitations

Given the lack of studies, we did not calculate the sample size a priori. A posteriori, from the total PSQI score obtained, for a power of 90% and a significance level of 0.05, a sample size of 45 participants it should be required. We have studied a limited number of patients, but with a nice prospective design, and intra-patient pre-post comparisons. In addition, actigraphy, even if its usefulness is demonstrated in sleep disorders assessment^14^, is not the gold standard to assess sleep, lacking accurate measurements of sleep architecture by electroencephalogram. On the other hand, contrarily to polysomnography (PSG), actigraphy is very easy to use, allows consecutive days recording and gives information about patient’s PA.

Finally, we did not formally exclude underlying sleep disorders by PSG. However, our patients were not obese and did not complain of typical obstructive sleep apnea symptoms.

In conclusion, PR does not improve SE nor subjective sleep quality in COPD patients. However, SE improved significantly in the poor sleeper subgroup (SE < 85%), whereas the PSQI remained unchanged in these patients. Further studies are needed to better characterize the origin of sleep disturbances in COPD and the potential benefit of some medications or non-pharmacologic interventions (e.g. cognitive behavioral therapy)^3^.

## Data Availability

The datasets used and analyzed during the current study are available from the corresponding author on reasonable request.
